# A Text-Book of Diseases of Women

**Published:** 1902-01

**Authors:** 


A Text-Book of Diseases of Women. By Charles B. Penrose, M. D., Ph. D., formerly Professor of Gynecology in the University of Pennsylvania. Fourth edition, revised. Octavo volume of 539 pages, handsomely illustrated. Philadelphia and London: W. B. Saunders & Co. 1901. Cloth, $3.75 net.

This book was intended primarily for the use of medical students, but it has been found to meet the needs of the practicing physician quite as well as it does those of the undergraduate. In the University of Pennsylvania it is used almost to the exclusion of every other work on gynecology. It is a complete practical textbook from which both physicians and students may readily instruct themselves in the best approved methods of diagnosis and treatment employed in modern gynecology. No one except a specialist has need for a more elaborate treatise.

To avoid confusing those who consult the book for guidance, the author has in almost every instance recommended but one plan of treatment, and that the treatment he has himself found to be best. The advantage of this can be readily appreciated.

The present edition is the result of a complete revision. It contains more matter than any of its predecessors and a number of new illustrations. R.

